# Quartz-Enhanced Photoacoustic Spectroscopy Sensor with a Small-Gap Quartz Tuning Fork

**DOI:** 10.3390/s18072047

**Published:** 2018-06-27

**Authors:** Yu-Fei Ma, Yao Tong, Ying He, Jin-Hu Long, Xin Yu

**Affiliations:** National Key Laboratory of Science and Technology on Tunable Laser, Harbin Institute of Technology, Harbin 150001, China; tongyao9505@163.com (Y.T.); hearkenyi@hit.edu.cn (Y.H.); ljh65923@163.com (J.-H.L.); yuxin0306@hit.edu.cn (X.Y.)

**Keywords:** QEPAS, small-gap QTF, gas sensor, COMSOL

## Abstract

A highly sensitive quartz-enhanced photoacoustic spectroscopy (QEPAS) sensor based on a custom quartz tuning fork (QTF) with a small-gap of 200 μm was demonstrated. With the help of the finite element modeling (FEM) simulation software COMSOL, the change tendency of the QEPAS signal under the influence of the laser beam vertical position and the length of the micro-resonator (mR) were calculated theoretically. Water vapor (H_2_O) was selected as the target analyte. The experimental results agreed well with those of the simulation, which verified the correctness of the theoretical model. An 11-fold signal enhancement was achieved with the addition of an mR with an optimal length of 5 mm in comparison to the bare QTF. Finally, the H_2_O-QEPAS sensor, which was based on a small-gap QTF, achieved a minimum detection limit (MDL) of 1.3 ppm, indicating an improvement of the sensor performance when compared to the standard QTF that has a gap of 300 μm.

## 1. Introduction

Quartz-enhanced photoacoustic spectroscopy (QEPAS) is a sensitive gas sensing technique that was invented in 2002 [1]. As an innovation based on traditional photoacoustic spectroscopy (PAS), QEPAS utilizes a millimeter-sized quartz tuning fork (QTF) as an acoustic transducer instead of a wide-band microphone employed in PAS. With merits including high sensitivity, compactness, fast response, and a large dynamic range, QEPAS has found applications in the detection of numerous trace gases [2,3,4,5,6,7,8].

The performance of a QEPAS sensor system is correlated with the measured signal value. Therefore, it is necessary to maximize the signal. An improvement of the QEPAS signal can be achieved by an increase in optical excitation power. In Ma et al. [9], a quantum cascade laser (QCL) was used as the excitation because the output power can reach ~1000 mW. An erbium-doped fiber amplifier (EDFA) with an output power of ~1200 mW was utilized to amplify the output laser in Wu et al. and Ma et al. [10,11]. In Lassen et al. [12], an off-beam QEPAS sensor using a pulsed nanosecond mid-infrared optical parametric oscillator with an average output power of approximately 400 mW was demonstrated. Additionally, an optimization of the dimensions of the QTF and the structure of the acoustic detection module could also improve QEPAS sensor performance [13,14,15,16,17]. 

QTF, a crystal component, is produced by photolithographic and chemical etching techniques [18]. Historically, QTF was used to provide the clock rate in crystal watches, timers, and electronic circuits. The unique acoustic quadrupole structure of a QTF provides an excellent immunity to environment interference [19]. With a U-shape geometry, a QTF consists of two quartz fork tines and a hollow fork valley. The acoustic wave causes resonance vibration, thereby accumulating the energy in the QTF. Standard commercial QTFs possess a resonance frequency, *f*_0_, of 32.768 kHz and a Q-factor of ~10,000 in standard atmosphere pressure. The width of the fork valley, the so-called gap, determines the space for acoustic wave generation. Traditional QTFs with a gap of 300 μm cannot satisfy the requirement when the excitation beam size exceeds the gap dimension. Therefore, adjusting the size of the QTF is a method to improve sensor performance [20,21]. In Borr et al. [21], a custom-made QTF with a 1 mm-wide prong spacing was used in terahertz (THz) spectral region detection. As the big beam waist diameter of the THz laser is larger than ~400–500 μm, this results in blockage of laser beam radiation by the times of a commercial QTF [18]. Nevertheless, in the near-infrared range, the diameter of the laser beam is far smaller than that in the THz spectral region.

**Figure 1:**
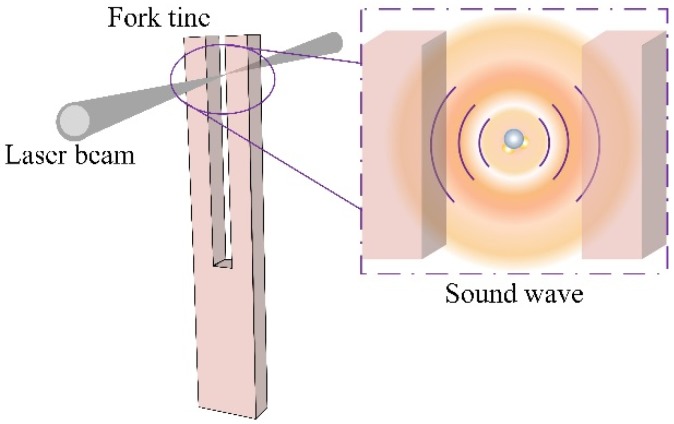
The schematic diagram of a quartz tuning fork (QTF), the laser excitation beam, and the generated sound wave.

The laser beam passes through the fork valley and excites the gas molecules. Notably, the sound wave generated by the reaction between the laser beam and the gas molecules can be viewed as spherical wave oscillation. Figure 1 shows the schematic diagram of the laser beam, a QTF, and the associated sound wave. The spherical wave propagation decreases with the cube of the distance, which suggests that the energy of the sound wave will diminish rapidly as the distance from the generation source point of the sound wave increases. From this perspective, employing a small-gap QTF can avoid energy losses and therefore improve the signal level of the QEPAS sensor.

In this paper, the improvement of a QEPAS sensor detection sensitivity using a QTF with a small-gap of 200 μm was demonstrated for the first time. Firstly, on the basis of the finite element modeling (FEM) of COMSOL software, a simulation of the optimal vertical position with respect to the QTF and the length of a micro-resonator was presented. Subsequently, water vapor (H_2_O) with an absorption line located at 7168.4 cm^−1^ was chosen as the target. Experimental results agreed well with the theoretical simulations and an improvement of QEPAS performance using the small-gap QTF was achieved.

## 2. Experimental Setup

**Figure 2:**
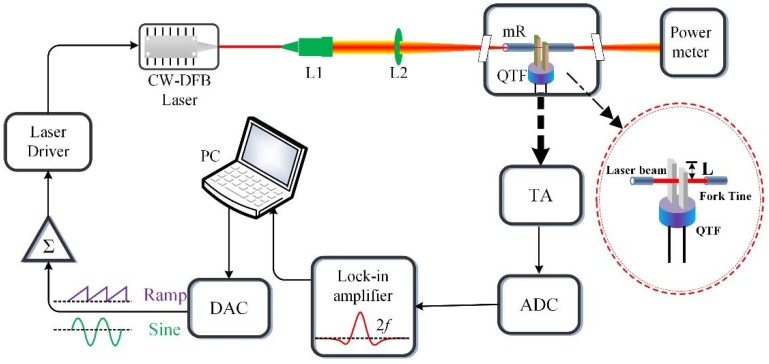
Schematic of the quartz-enhanced photoacoustic spectroscopy (QEPAS) sensor system.

The basic design of the experimental setup is shown in Figure 2. A distributed feedback (DFB) diode laser with an output wavelength of 1.39 μm was employed. The jitter of the diode laser output power was less than 1%. The laser beam quality was measured and the beam quality factor M^2^ was found to be 1.03 at an output power of 10.5 mW when the H_2_O absorption line was targeted. The scanning of wavelengths was completed by adding a voltage ramp as a current driver. Additionally, a sinusoidal signal with a frequency of *f*_0_/2 was applied to modulate the laser. After collimation, the diode laser beam was focused into the QTF valley. The laser beam size at the focal point was ~42 μm when a lens with a focal length of 60 mm was used. Because the generated piezoelectric current signal was too weak to detect directly, a custom-made transimpedance amplifier (TA) with a feedback resistance of 10 MΩ was used to amplify the QTF signal. The voltage signal was delivered to a lock-in amplifier, where it was demodulated into a second harmonic signal (2*f*). The integration time for the QEPAS sensor was 1 s. A serial port (RS232) provided an output for the data flow from the lock-in amplifier to a personal computer (PC). A custom-made, small-gap QTF was employed in this research. The dimensions of the QTF are shown in Table 1.

## 3. Theoretical Simulations

**Figure 3:**
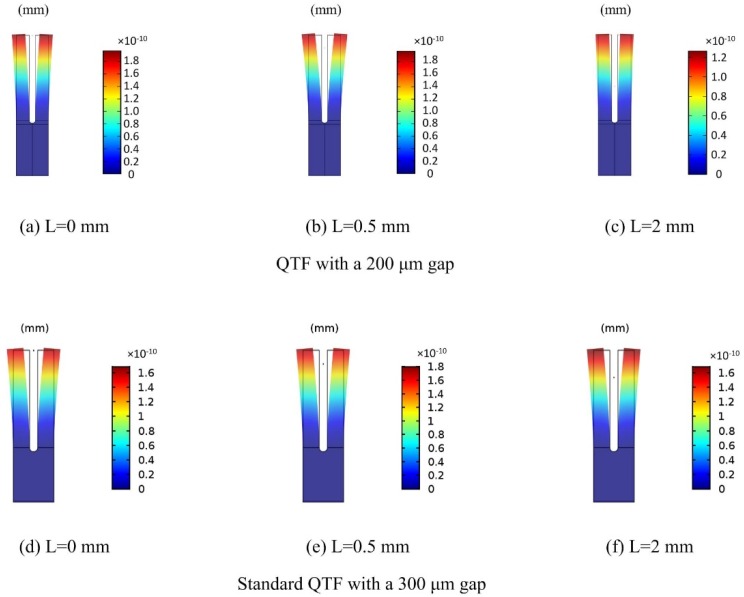
Calculated deformation with various acoustic wave excitation positions (L) for two different QTFs: (**a**–**c**) for the QTF with a 200 μm gap; (**d**–**f**) for the standard QTF with a 300 μm gap.

In this section, an acoustic generation module formed by the QTF and a cone laser beam was simulated using the FEM COMSOL software. Invoking the acoustic module and structural mechanics module, a finite element model was constructed to calculate the vibration modes in varying conditions [22]. An illustration in Figure 2 depicts the definition of L, which represents the distance between the unfixed top of the fork tine and the laser beam. The deformation of the QTF with the laser beam radiating at L = 0 mm, L = 0.5 mm, and L = 2 mm is shown in Figure 3. The black solid lines represent the inherent shape of the QTF while the multicolor region represents the deformation caused by the acoustic wave. A color bar is used to represent the deformation value. It can be seen that a maximal pendulum deflection occurred at the condition of L = 0.5 mm. For comparison, a standard QTF with a gap of 300 μm is also presented in Figure 3.

**Figure 4:**
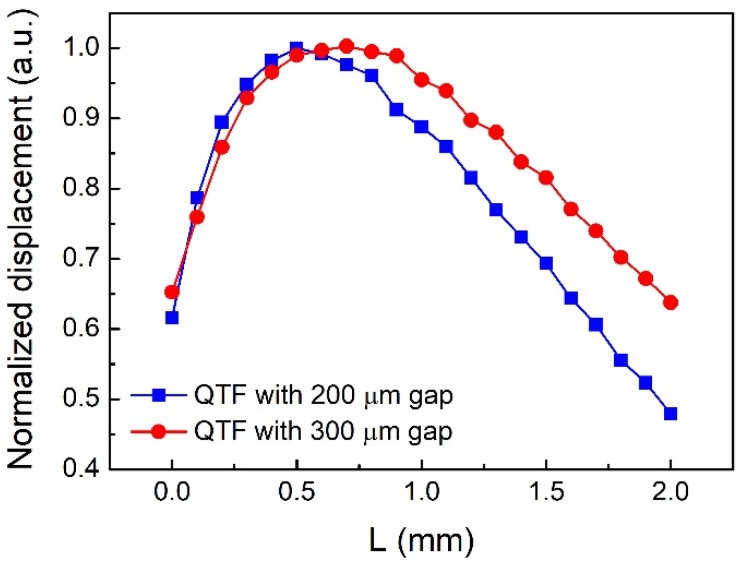
Normalized calculated displacement as a function of vertical height (L) for two different QTFs.

The simulated QEPAS normalized displacement with various values of L for two different QTFs is shown in Figure 4. The calculated range was 0–2 mm with a 0.1 mm step size. As the diode laser beam moved down relative to the QTF, the normalized signal amplitude increased rapidly in the range of 0–0.5 mm and kept decreasing within the range of 0.5–2 mm for the QTF with a 200 μm gap. The optimum vertical position was L = 0.5 mm based on the simulated results. For the standard QTF with a 300 μm gap, the optimum vertical position L was 0.7 mm. The calculated maximum displacement for the small-gap QTF with 200 µm gap and the standard QTF with 300 µm gap were 1.96 × 10^−10^ mm and 1.82 × 10^−10^ mm, respectively.

**Figure 5:**
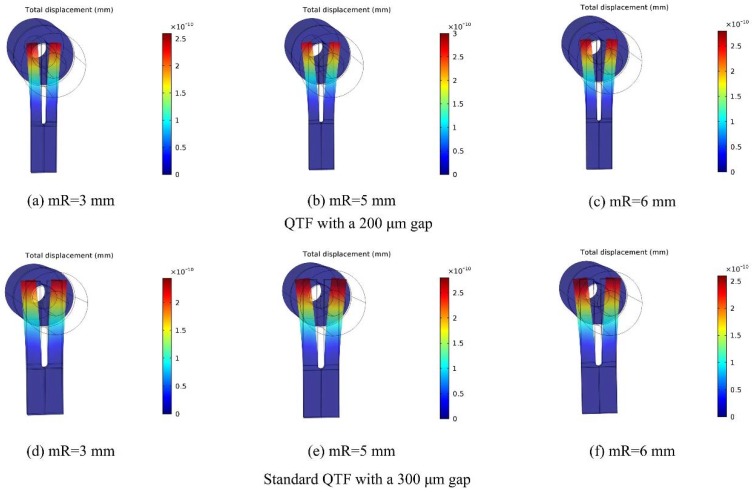
Calculated displacement with various micro-resonators (mRs) for two different QTFs: (**a**–**c**) for the QTF with a 200 μm gap; (**d**–**f**) for the standard QTF with a 300 μm gap.

**Figure 6:**
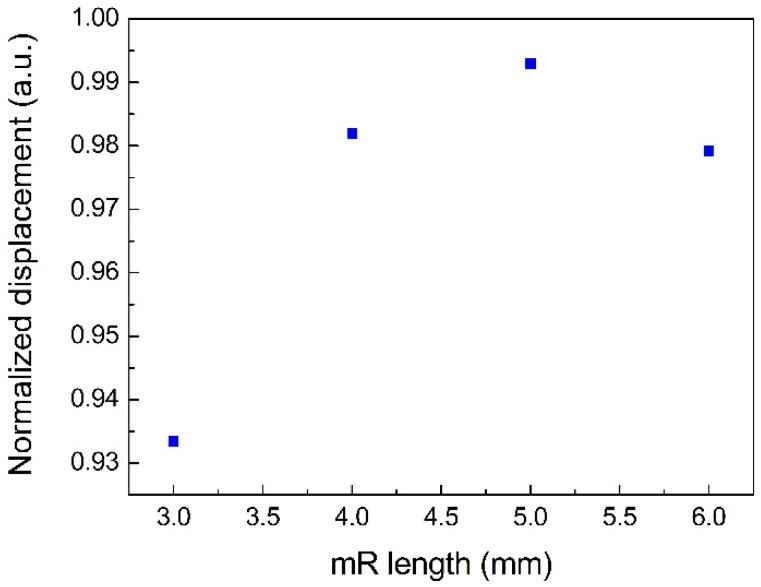
Calculated normalized displacement as a function of mR length.

It was demonstrated that two metal micro-resonators (mRs) could improve the QEPAS signal as a result of the acoustic coupling between the two mRs and the QTF architecture. In Dong et al. [23], the optimal length of mR was shown to be λs/4~λs/2 where λs was the sound wavelength. Therefore, the optimal length range of mR was calculated to be 2.6–5.2 mm with the sound velocity of 340 m/s and the resonance frequency of 32.768 kHz. In the following simulations, the dimensions of mR tubes were set with an inner diameter of 0.5 mm and an outer diameter of 1.27 mm, which was in agreement with the mRs in the laboratory. On the basis of the investigation by Dong et al. [23], in this research, the gap between the QTF surface and the end of the mRs was set to be 25 μm for a strong acoustic coupling. The displacement of the fork tines was calculated for mRs with the length of 3 mm, 4 mm, 5 mm, and 6 mm. The sound wave generated position was set at L = 0.5 mm for the QTF with a small gap of 200 μm. The simulated diagrams of 3 mm, 5 mm, and 6 mm are shown in Figure 5. A transparent column and a blue-filled column, which symbolized the mRs, were placed at the two sides of the QTF. It can be seen that a larger displacement was achieved with an mR of 5 mm. For the standard QTF with a 300 μm gap, the L was set to be 0.7 mm in the simulation. The calculated results are also presented in Figure 5 and the maximum displacement was obtained when the length of mR was 5 mm. The calculated normalized displacement with different mRs is shown in Figure 6. The enhancement effect of the mR from high to low was in the order of 5 mm, 4 mm, 6 mm, 3 mm.

## 4. Results and Discussion

**Figure 7:**
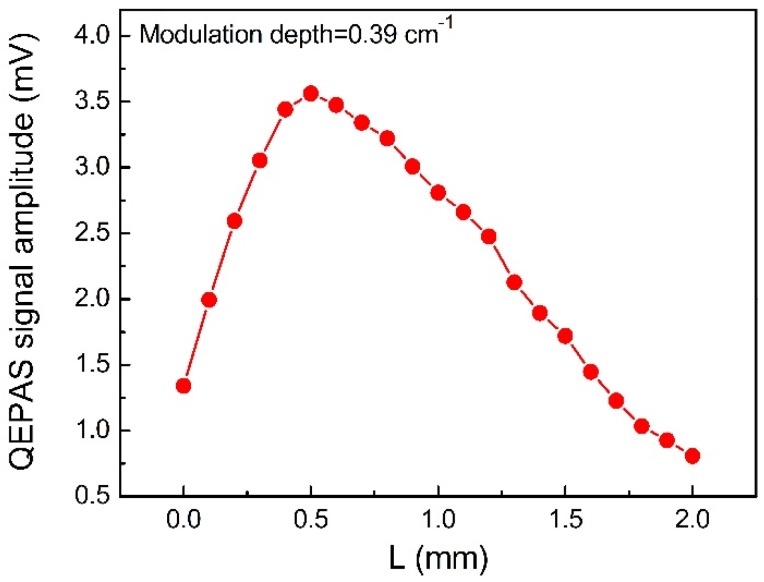
Experimental measured H_2_O-QEPAS signal amplitude as a function of the vertical height of the laser beam.

As mentioned above, water vapor (H_2_O) was selected as the target analyte and a QTF with a small-gap of 200 μm was adopted. The acoustic wave excitation position generated by the diode laser beam was optimized experimentally and the result is shown in Figure 7. The experiment was carried out with a laser wavelength modulation depth of 0.39 cm^−1^. The experimental results were often identical to the FEM simulations and the optimal position of L for the maximal signal amplitude was found to be 0.5 mm. Therefore, in the following experiments L = 0.5 mm was chosen.

**Figure 8:**
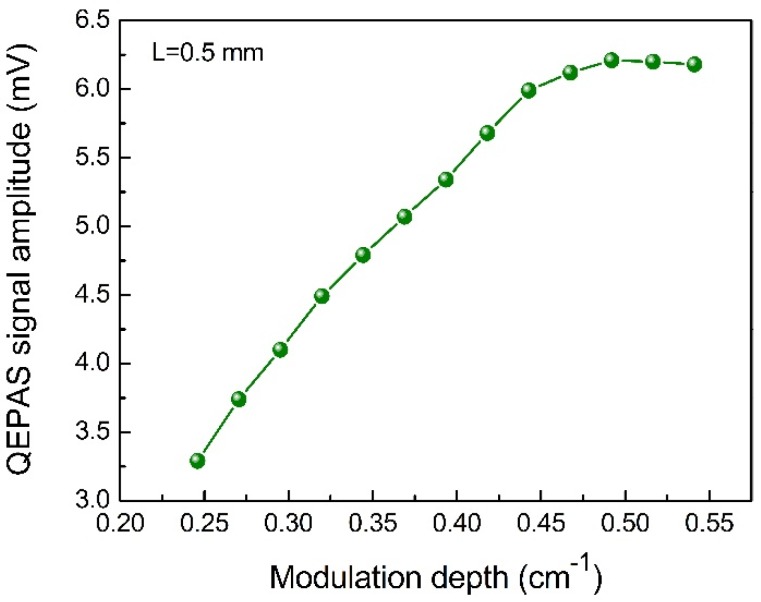
Experimental measured H_2_O-QEPAS signal amplitude as a function of modulation depth.

An optimization of the modulation depth was implemented to improve the 2*f* signal level. The laser modulation was completed by adding a voltage ramp with low frequency and a sinusoidal signal with a frequency of *f*_0_/2. The experimental results are shown in Figure 8. When the modulation depth was small, the modulation amplitude fell short of covering the H_2_O absorption line. Therefore, the QEPAS signal amplitude was weak. The H_2_O-QEPAS signal level increased with an increase of the modulation depth. The curve declined when the modulation depth was greater than 0.49 cm^−1^. Ultimately, the optimum modulation depth was determined to be 0.49 cm^−1^.

**Figure 9:**
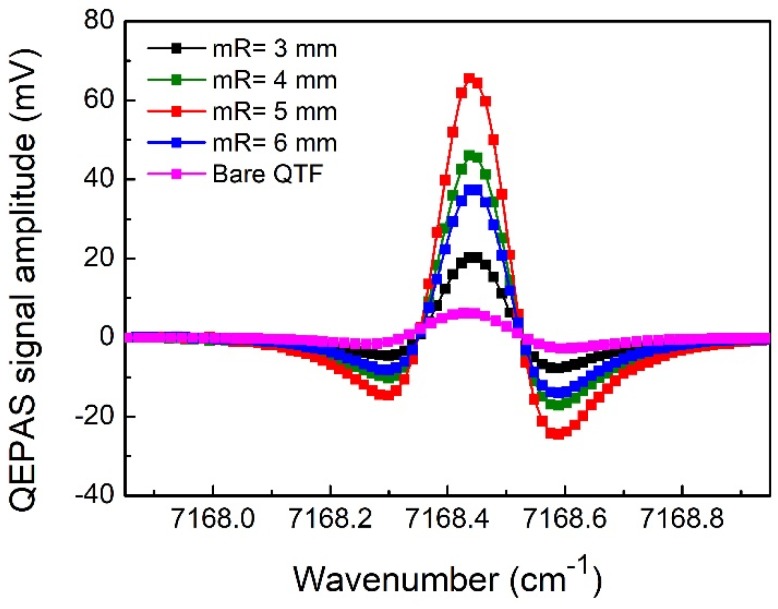
Experimental measured 2*f* QEPAS signals using mRs with different lengths

The range of 2.8~5.5 mm was the optimum length of the mR tubes, as mentioned before. The 2*f* signal measured following the addition of mRs of different lengths at the optimum modulation depth of 0.49 cm^−1^ is shown in Figure 9. The maximum signal level was achieved when the mR tubes with a length of 5.0 mm were used, followed by the lengths of 4 mm, 6 mm, and 3 mm. These results were consistent with those of the simulation. The maximal signal of 65.6 mV achieved an 11-fold amplification when compared to the signal generated by the bare small-gap QTF. The noise value was 29.6 μV, which was obtained by taking the standard deviation of signal points distant from the absorption peak. The integration time for the noise determination was the same as the signal measurement. With a water vapor concentration of 0.296% (with air temperature at ~13 °C), the minimum detection limit (MDL), which represents the detection performance of sensor, was calculated to be 1.3 parts per million (ppm). The calculated normalized noise equivalent absorption coefficient (NNEA) was 1.42 × 10^−^^8^ cm^−^^1^W/√Hz.

When an mR with a length of 4 mm was used, a signal enhancement of 7.5 times was obtained, resulting in an MDL of 1.85 ppm and a NNEA of 2.02 × 10^−^^8^ cm^-1^W/√Hz for this H_2_O-QEPAS sensor using a QTF with a small-gap of 200 μm. For comparison, the MDL and NNEA for the H_2_O-QEPAS sensor using a standard QTF with a gap of 300 μm and an mR of 4 mm and a laser power of 12.6 mW were 5.9 ppm and 7.73 × 10^−8^ cm^-1^W/√Hz, respectively [24]. This significant improvement can be explained as follows. On one hand, the small-gap QTF, with a lower energy loss of diffusion, allowed the acoustic wave energy to push the fork tines more efficiently. On the other hand, a decrease of the fork tine mass compared to that of a commercial QTF may lead to a larger displacement. 

## 5. Conclusion

In conclusion, a sensitive QEPAS sensor employing a custom small-gap QTF as an acoustic wave transducer was demonstrated in this paper for the first time. Firstly, on the basis of the finite element modeling (FEM) software COMSOL, a theoretical model was built to predict the tendency of the QEPAS signal when both the position of the diode laser beam and the length of the mR were changed. Water vapor was chosen as the target analyte. Experiments were carried out and the experimental results matched well with those of the simulation. The maximum 2*f* signal level was obtained when the laser beam was 0.5 mm away from the end of the tine and the mR length was 5 mm. The optimized laser wavelength modulation depth was found to be 0.49 cm^−1^. Furthermore, a 5 mm mR yielded a detection sensitivity enhancement factor of 11 with respect to the bare QTF. Finally, an MDL of 1.3 ppm was achieved at a 1 s integration time. It can be seen that a small-gap QTF increases the acoustic wave energy strength between the fork tines and, therefore, enhances the QEPAS sensor performance. The detection limit can be further improved when a QTF with an optimum gap is adopted.

## Figures and Tables

**Table 1:** Dimensions and parameters of the small-gap QTF.

	Parameter	Unit	Small-Gap QTF
**QTF**	Measured frequency *f*_0_	Hz	32,754.27
	Gap	μm	200
	Q factor	--	8179
**Fork tine**	Length	mm	3.42
	Thickness	mm	0.44
	Width	mm	0.3
